# The association of genetic polymorphisms with nonalcoholic fatty liver disease in a longitudinal study

**DOI:** 10.1186/s12876-020-01469-8

**Published:** 2020-10-15

**Authors:** Goh Eun Chung, Eunsoon Shin, Min-Sun Kwak, Jong In Yang, Jong-Eun Lee, Eun Kyung Choe, Jeong Yoon Yim

**Affiliations:** 1 Department of Internal Medicine, Gangnam Healthcare Center, Seoul National University Hospital, 39FL., Gangnam Finance center 737, Yeoksam-Dong, Gangnam-Gu, Seoul, 135-984 South Korea; 2 DNA Link, Inc., Seoul, South Korea; 3 Healthcare Research Institute, Seoul National University Hospital, Healthcare System Gangnam Center, Seoul, South Korea

**Keywords:** Nonalcoholic fatty liver disease, Genome-wide association study, Single-nucleotide polymorphism

## Abstract

**Background:**

Several genetic variants are known to be associated with nonalcoholic fatty liver disease (NAFLD). We aimed to evaluate the longitudinal associations between genetic variants and NAFLD.

**Methods:**

We performed a genome-wide association study (GWAS) in Korean individuals who underwent repeated health check-ups. NAFLD was defined by ultrasonography and exclusion of secondary causes.

**Results:**

The subjects had a median age of 50.0 years, and 54.8% were male. The median follow-up duration was 39 months. Among the 3905 subjects without NAFLD at baseline, 874 (22.4%) subjects developed NAFLD, and among the 1818 subjects with NAFLD at baseline, NAFLD regressed in 336 (18.5%) subjects during the follow-up period. After adjusting for age, sex and body mass index, no single-nucleotide polymorphism (SNP) passed Bonferroni correction for genome-wide significance in the development or regression of NAFLD. Among the SNPs that passed the genome-wide suggestiveness threshold (*p* = 1E-04) in the discovery set in the GWAS, only 1 SNP (rs4906353) showed an association with the development of NAFLD, with marginal significance in the validation set (*p*-value, discovery set = 9.68E-5 and validation set = 0.00531).

**Conclusions:**

This exploratory study suggests that longitudinal changes in NAFLD are not associated with genetic variants in the Korean population. These findings provide new insight into genetic mechanisms in the pathogenesis of NAFLD.

## Background

Nonalcoholic fatty liver disease (NAFLD) is one of the most common chronic liver diseases in the world, with an increasing prevalence of up to 20–30% in the general population [1]. While the majority of NAFLD patients follow a relatively benign clinical course, with simple hepatic steatosis or mild nonalcoholic steatohepatitis, approximately 25% of patients may experience progression to advanced liver disease and have increased liver-related mortality [2–4]. In Asia, the annual incidence of hepatocellular carcinoma and the overall mortality in patients with NAFLD are increasing, with few effective treatments [5].

NAFLD development is driven by environmental factors, but it also requires genetic susceptibility [6, 7]. Previous genome-wide association studies (GWASs) have identified a variety of genes and single-nucleotide polymorphisms (SNPs) that confer susceptibility to NAFLD [8]. Notably, the patatin-like phospholipase domain-containing protein 3 (*PNPLA3*) gene, encoding the isoleucine to methionine variant at protein position 148 (I148M), on chromosome 22 was strongly associated with an increased liver fat content [9]. A systematic review showed that the rs738409 GG genotype was associated with not only liver fat accumulation but also susceptibility to more aggressive disease traits in multiple ethnic groups [10]. In studies based on Asian populations, there were significant associations of *PNPLA3* with the occurrence and severity of NAFLD [11–13].

Although recent GWASs have identified a large number of genetic variants that confer susceptibility to fatty liver, they have been conducted in a cross-sectional manner that typically involves the measurement of clinical traits at a single point in time. Therefore, subjects who are likely to be affected by fatty liver in the future may be regarded as controls at the time of data collection, and the causal relationship cannot be evaluated. To address this issue, we aimed to evaluate the longitudinal association between NAFLD and SNPs in the Korean population.

## Methods

### Study population

**Fig. 1:**
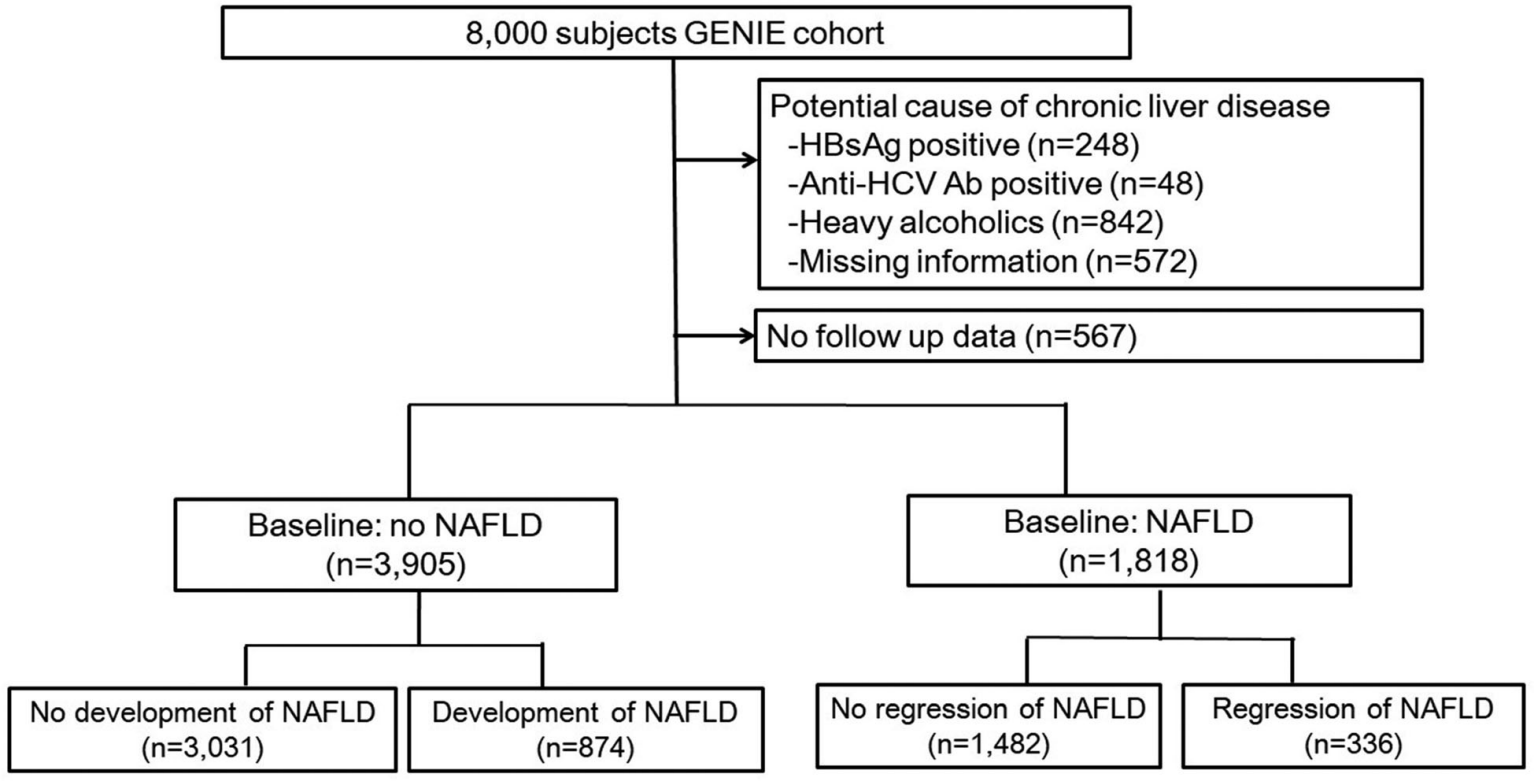
Flow diagram of the subjects evaluated in the study

In this cohort study, we conducted a post hoc analysis of the previously reported GENIE cohort [12, 14]. Briefly, from January 2014 to December 2014, 8000 people donated blood samples while participating in a routine comprehensive health check-up program at the Seoul National University Hospital Gangnam Center after providing informed consent. We excluded subjects with significant alcohol intake (> 20 g/day for males and > 10 g/day for females), subjects positive for hepatitis B virus and hepatitis C virus, and subjects with missing information. As a result, a total of 6290 subjects were initially included in the Gangnam NAFLD cohort [12]. Additionally, we excluded 567 subjects who did not attend any voluntary follow-up health check-ups. Ultimately, 5723 subjects were included for analysis (Fig. 1). The median follow-up duration was 39 months (interquartile range, 25–55 months). This study protocol was performed in accordance with the Declaration of Helsinki and approved by the Ethics Committee of the Seoul National University Hospital with a waiver of informed consent (No. 1705–113-855).

### Clinical and laboratory assessments

The methods employed in this study have been described in detail elsewhere [15]. Briefly, each participant completed a structured self-reported questionnaire including past medical history and an anthropometric assessment. The body mass index (BMI) was calculated as body weight (in kilograms) divided by the squared height (in meters). Hypertension was defined as systolic blood pressure ≥ 140 mmHg or diastolic blood pressure ≥ 90 mmHg or use of antihypertensive medication. Diabetes mellitus was defined as either a fasting serum glucose ≥126 mg/dL or the use of antidiabetic medication. The laboratory and radiologic tests were performed on the same day. All laboratory tests were performed using standard methods. The laboratory examinations included serum aspartate aminotransferase (AST), alanine aminotransferase (ALT), total cholesterol, triglycerides, high-density lipoprotein (HDL) cholesterol, fasting glucose, hepatitis B surface antigen and antibodies to hepatitis C virus. Elevation of liver enzyme levels was defined as elevated serum ALT above the strict cutoff point based on the updated definition (ALT > 30 IU/L for males and > 19 IU/L for females) from Prati et al. [16] We then classified the subjects into 4 groups based on these normal values of ALT: 1) normal, those with normal values at both baseline and follow-up; 2) elevated, those with normal values at baseline and abnormal values at follow-up; 3) reduced, those with abnormal values at baseline and normal values at follow-up; and 4) persistent, those with abnormal values at both baseline and follow-up.

The diagnosis of NAFLD was based on the ultrasonography findings (Acusion, Sequoia 512, Siemens, Mountain View, CA), as determined by experienced radiologists who were unaware of the clinical details of the subjects. The same board-certified radiologists performed ultrasonography at the baseline and follow-up exams. Follow-up evaluations of hepatic ultrasonography were similarly conducted with the standard protocol and equipment. The sonographic features of fatty liver were based on standard criteria, including hepatorenal contrast, parenchymal brightness, deep beam attenuation and bright vessel walls [17, 18]. We then classified subjects into 4 groups according to fatty liver status: 1) none, those without fatty liver at both baseline and follow-up; 2) developed, those without fatty liver at baseline and with fatty liver at follow-up; 3) regressed, those with fatty liver at baseline and without fatty liver at follow-up; and 4) persistent, those with fatty liver at both baseline and follow-up.

### Genome-wide genotyping and quality control

For genomic DNA separation, we used the Affymetrix Axiom™ KORV1.0–96 Array (Thermo Fisher Scientific, Santa Clara, CA, USA) based on the manufacturer’s protocol. Approximately 200 ng of genomic DNA was initially amplified, and genotype data were generated using the Korean-Chip, that was designed by the National Institute of Health Genome Science Center, Korea (4845–301, 3000–3031). Genotyping was performed by DNA Link, Inc., with standard systematic quality control procedures. The PLINK software (version 1.9, Free Software Foundation Inc., Boston, MA, USA) was used for quality control. Samples that met one of the following criteria were removed: (i) sample call rate ≤ 97%, (ii) sex inconsistency, or (iii) related or cryptically related individuals (kinship coefficient > 0.0875). SNPs were filtered if (1) the call rate was < 95%, (2) the minor allele frequency was ≤1%, or (3) there was a significant deviation from Hardy-Weinberg equilibrium for the control (*P* < 0.0001). In addition, visual inspection of the cluster plot excluded SNPs that were likely to have false-positive associations due to incorrect clustering.

### Statistical analysis

The outcome of this study was the development and regression of NAFLD. We used the chi-squared test or Fisher’s exact test for categorical variables and Student’s t-test for continuous variables. Regression analysis with Cox proportional hazard models was used to analyze the adjusted hazard ratio (HR) and 95% confidence interval (CI) for the development or regression of NAFLD and the elevation or reduction of ALT levels after controlling for potential confounders, including age, sex and BMI, using SAS statistical software, version 9.1.3 (SAS Institute Inc., Cary NC). Regional plotting was performed with the LocusZoom program (http://locuszoom.org).

The registered samples were divided into two sets based on the time of enrollment. Samples collected from January 2014 to October 2014 were used as the discovery set, and those enrolled in the following period composed the validation set. The intention was to re-evaluate and verify the results in the replication set any SNPs that had *P*-values of less than 5 × 10^− 8^ in the discovery set. However, since no SNPs had *P*-values less than 5 × 10^− 8^ in the discovery set, rather than applying Bonferroni’s correction criteria, we selected SNPs that had a less stringent *P*-value cutoff (1E-04). *P*-values less than 0.05 were considered significant in the validation set.

## Results

### Study population characteristics

The mean age of the study subjects was 50.0 ± 10.1 years, and 54.8% were male. Table 1 shows the clinical characteristics of the study population. The validation set had a larger proportion of males and higher systolic and diastolic blood pressure, BMI, ALT, total cholesterol and fasting glucose (*p* < 0.05). Among the 4117 subjects in the discovery set, 643 (22.1%) developed NAFLD, and 240 (18.5%) showed regression of NAFLD; among 1605 subjects in the validation set, 231 (23.1) developed NAFLD, and 96 (18.5) showed regression of NAFLD.

**Table 1:** Baseline characteristics of the study population

	Discovery set (*N* = 4201)	Validation set (*N* = 1522)	*P*-Value
Age (years)	49.7 ± 10.2	49.3 ± 9.8	0.158
Male, *n* (%)	2259 (53.8)	878 (57.7)	0.009
Diabetes mellitus, *n* (%)	194 (4.6)	77 (5.1)	0.487
Hypertension, *n* (%)	675 (16.1)	256 (16.8)	0.496
Systolic blood pressure (mmHg)	114.6 ± 13.2	115.6 ± 13.0	0.010
Diastolic blood pressure (mmHg)	75.1 ± 10.2	76.3 ± 9.9	< 0.001
Body mass index (kg/m^2^)	22.9 ± 3.0	23.2 ± 3.0	0.003
Waist circumference (cm)	82.2 ± 8.7	82.1 ± 8.7	0.749
AST (IU/L)	22.6 ± 9.5	23.0 ± 10.4	0.198
ALT (IU/L)	22.3 ± 15.0	23.5 ± 17.0	0.017
Total cholesterol (mg/dL)	192.6 ± 33.7	194.6 ± 34.0	0.046
Triglyceride (mg/dL)	105.5 ± 68.2	107.7 ± 72.0	0.313
High-density lipoprotein cholesterol (mg/dL)	53.8 ± 12.0	53.9 ± 12.0	0.637
Fasting glucose (mg/dL)	97.5 ± 15.0	99.3 ± 19.3	0.001
Development of NAFLD, *n* (%)	643 (22.1)	231 (23.1)	0.554
Regression of NAFLD, *n* (%)	240 (18.5)	96 (18.5)	0.989

Data are shown as the mean ± SD

*AST* aspartate aminotransferase, *ALT* alanine aminotransferase, *NAFLD* nonalcoholic fatty liver disease

Among the 5723 subjects at baseline, 3905 (68.2%) showed no NAFLD, and 1818 (31.8%) showed NAFLD. The characteristics of the study population are described in Table 2. Among subjects without fatty liver at baseline, 874 (22.4%) subjects developed NAFLD during the follow-up period. Most of the demographic, anthropometric, and laboratory variables (including sex, BMI, waist circumference, AST, ALT, triglycerides, HDL-cholesterol, and fasting glucose) were less metabolically favorable in subjects who developed NAFLD than in those who did not develop NAFLD (*P* < 0.001). Among subjects with NAFLD at baseline, NAFLD regressed in 336 (18.5%) subjects during the follow-up period. Comparisons of baseline characteristics revealed that subjects with regression of NAFLD were older and had lower blood pressure, BMI, WC, and levels of AST, ALT, triglycerides, HDL-cholesterol and fasting glucose (Table 2).

**Table 2:** Relationship between patient characteristics and the development or regression of nonalcoholic fatty liver disease

	No development of NAFLD (*N* = 3031)	Development of NAFLD (*N* = 874)	*P*-Value	No regression of NAFLD (*N* = 1482)	Regression of NAFLD (*N* = 336)	*P*-Value
Age (years)	48.3 ± 10.3	50.2 ± 9.6	< 0.001	51.2 ± 9.5	52.7 ± 10.3	0.012
Male, *n* (%)	1206 (39.8)	505 (57.8)	< 0.001	1184 (79.9)	242 (72.0)	0.002
Diabetes mellitus, *n* (%)	44 (1.5)	52 (5.9)	< 0.001	151 (10.2)	24 (7.1)	0.087
Hypertension, *n* (%)	308 (10.2)	150 (17.2)	< 0.001	397 (26.8)	76 (22.6)	0.116
Systolic blood pressure (mmHg)	112.0 ± 12.9	116.7 ± 13.3	< 0.001	119.3 ± 12.2	116.2 ± 12.7	< 0.001
Diastolic blood pressure (mmHg)	73.0 ± 9.9	76.7 ± 10.0	< 0.001	79.5 ± 9.4	76.2 ± 9.4	< 0.001
Body mass index (kg/m^2^)	21.6 ± 2.4	23.8 ± 2.5	< 0.001	25.2 ± 2.7	23.6 ± 2.6	< 0.001
Waist circumference (cm)	77.9 ± 7.4	84.7 ± 6.9	< 0.001	88.9 ± 7.1	84.8 ± 7.2	< 0.001
AST (IU/L)	21.3 ± 8.8	22.3 ± 7.7	0.001	25.9 ± 12.1	22.5 ± 7.6	< 0.001
ALT (IU/L)	18.1 ± 11.4	23.2 ± 13.5	< 0.001	31.6 ± 20.1	22.7 ± 11.4	< 0.001
Cholesterol (mg/dL)	191.4 ± 31.8	197.8 ± 35.3	< 0.001	194.7 ± 36.3	190.2 ± 34.7	0.035
Triglycerides (mg/dL)	82.6 ± 44.8	117.9 ± 64.6	< 0.001	146.2 ± 91.8	112.2 ± 57.6	< 0.001
HDL cholesterol (mg/dL)	57.6 ± 12.2	51.8 ± 10.7	< 0.001	47.9 ± 9.2	51.8 ± 11.1	< 0.001
Fasting glucose (mg/dL)	93.4 ± 11.8	100.1 ± 14.9	< 0.001	105.7 ± 21.0	100.6 ± 16.4	< 0.001
Elevation in ALT levels, *n* (%)	332/2091 (15.9)	178/629 (28.3)	< 0.001			
Reduction in ALT levels, *n* (%)				214/454 (47.1)	69/92 (75.0)	< 0.001

Data are shown as the mean ± SD

*NAFLD* nonalcoholic fatty liver disease, *AST* aspartate aminotransferase, *ALT* alanine aminotransferase, *HDL* high-density lipoprotein

### Genome-wide association analysis of NAFLD development

**Fig. 2:**
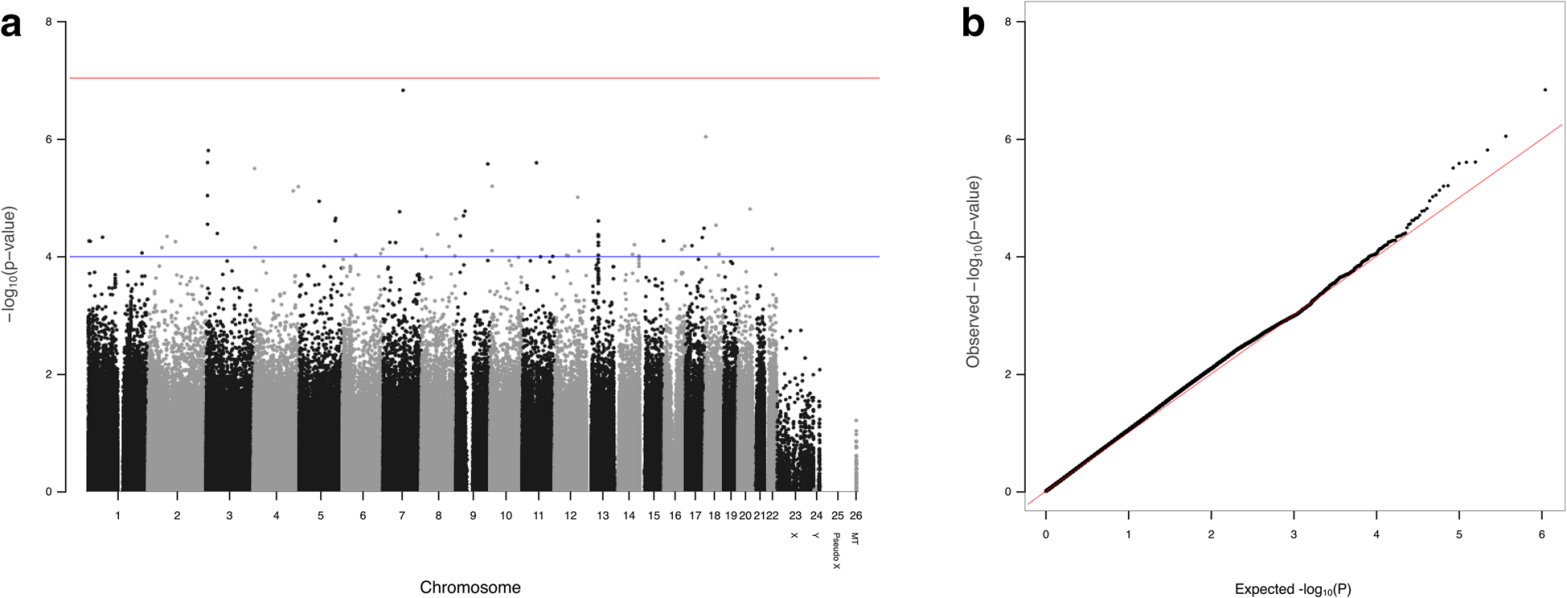
Manhattan plot of genome-wide association signals between SNPs and NAFLD (**a**) and a quantile–quantile plot (**b**) for the discovery set. In the Manhattan plot, the x-axis represents the SNP markers on each chromosome. The y-axis shows the -log10 *p*-value (logistic regression). The red horizontal line indicates the Bonferroni-adjusted significance threshold (5 × 10^−8^), and the blue horizontal line represents the genome-wide suggestiveness threshold (*p =* 1E-04). In the quantile–quantile plot, the x-axis and y-axis indicate the negative log-scale of the expected *p*-values for each SNP and the negative log-scale of the observed *p*-values, respectively. A straight line indicates the expected results under Hardy-Weinberg equilibrium

In the discovery set, an association analysis was conducted for the development of NAFLD using SNPs that passed quality control. After quality control, 546,592 autosomal SNPs remained for the association analysis. Quantile–quantile (QQ) plots generally showed little evidence of good agreement with statistical inflation, except for the tail part, where deviations are expected due to true association (Fig. 2).

We performed a GWAS for the development of NAFLD in the discovery set with significant *p*-values below 5 × 10^− 8^ as the threshold after adjusting for age, sex and BMI. Figure 2 also shows a Manhattan plot of the GWAS of the patients who developed NAFLD. The results showed that no SNP passed Bonferroni correction for genome-wide significance in the discovery set. Although no single common or rare variant reached genome-wide significance, we performed an exploratory analysis on the dataset of this GWAS of NAFLD development.

**Fig. 3:**
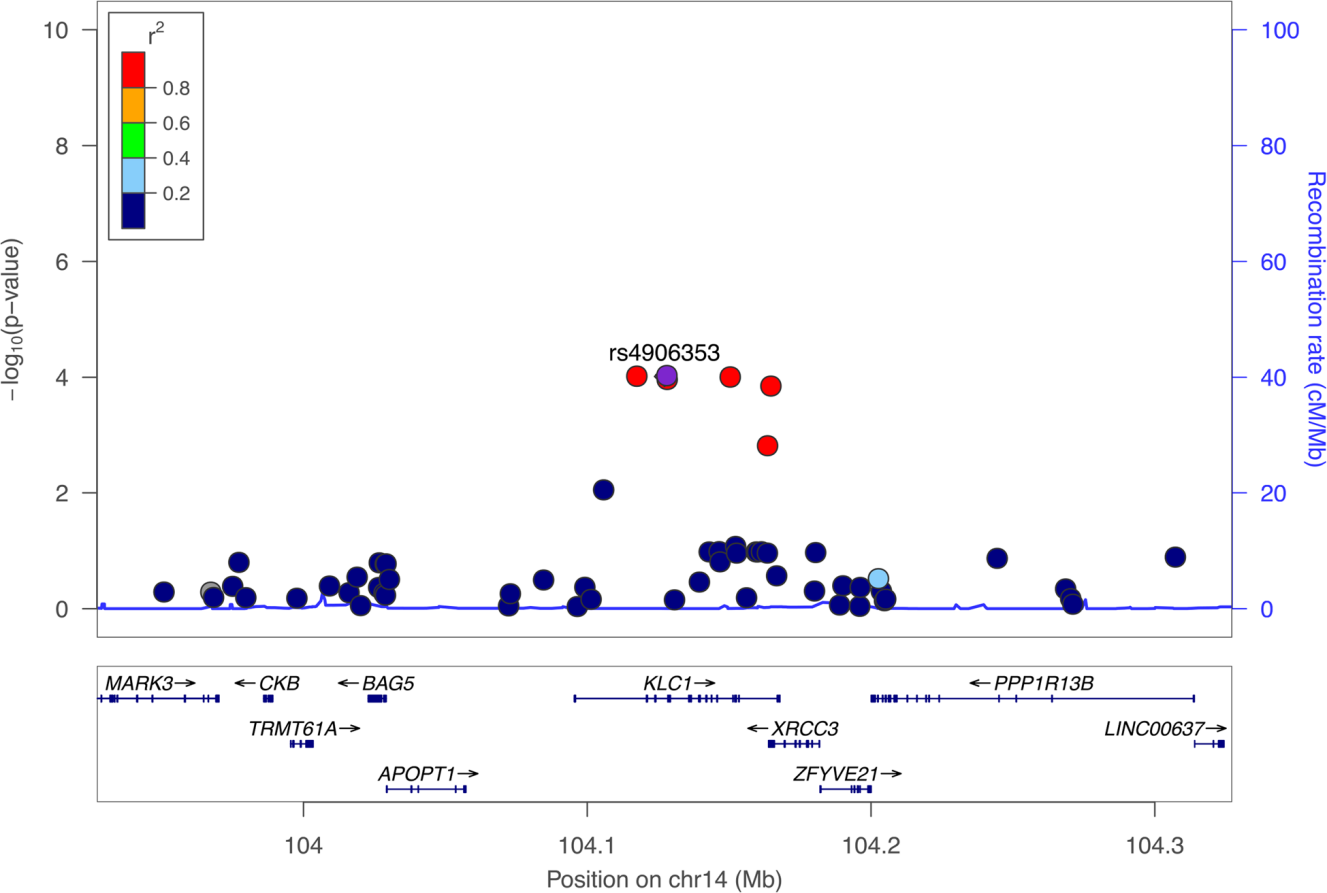
Regional association plot for chromosome 14. The purple diamonds indicate the associated SNPs according to joint analyses. Nearby SNPs are color-coded according to the level of linkage disequilibrium with the top SNP. The left y-axis shows the significance of the association based on the -log10 *p-*value (logistic regression), and the right y-axis shows the recombination rate across the region. Estimated recombination rates from the 1000 Genomes Project Asian database and the hg19 database [19] are plotted with the blue line to reflect the local linkage disequilibrium structure

Among the SNPs that passed the genome-wide suggestiveness threshold (*p* = 1E-04) in the discovery set of the GWAS, only 1 SNP (rs4906353) on 14 chromosomes showed an association with the development of NAFLD, with marginal significance in the validation set (*p*-value, discovery set = 9.68E-5 and validation set = 0.00531). The regional plot of chromosome 14 is provided in Fig. 3. The subjects with rs4906353 were found to have a decreased HR of developing NAFLD in multivariate logistic regression analysis after adjusting for age, sex, and BMI (adjusted HR 0.72, 95% CI 0.61–0.85 in the discovery set and HR 0.65, 95% CI 0.48–0.88 in the validation set, Table 3).

**Table 3:** SNP associated with the development of nonalcoholic fatty liver disease

					Discovery			Validation		
SNP	Chr	Position	Nearest Genes	Risk Allele	Risk allele Frequency	HR (95% CI)	*P*-value	Risk allele Frequency	HR (95% CI)	*P*-value
rs4906353	14	104,127,246	KCL1	T	0.030	0.72 (0.61, 0.85)	9.68E-5	0.026	0.65 (0.48, 0.88)	0.00531

*SNP* single-nucleotide polymorphism, *Chr* chromosome number, *HR* hazard ratio, *CI* confidence interval, *KCL* kinesin light chain

*P* values are adjusted for age, sex and body mass index. An additive genetic model was used

### Genome-wide association analysis of NAFLD regression

Next, we performed a GWAS for the regression of NAFLD in the discovery set, with significant *p*-values below 5 × 10^− 8^ as the threshold after adjusting for age, sex and BMI. Supplementary Fig. 1 shows a Manhattan plot for the cases of NAFLD regression. The Manhattan plot was drawn using data from the discovery set. Among the 80 SNPs that passed the genome-wide suggestiveness threshold in the discovery set of the GWAS, no SNPs showed a significant association in the validation set.

We also evaluated transaminase (ALT) levels as a surrogate quantitative biomarker for NAFLD disease activity. As a result, the top two SNPs that showed genetic significance with elevation or reduction of ALT levels, were not replicated in the validation set (data not shown).

## Discussion

Although many genetic variants have been associated with NAFLD, almost all the studies reported to date have used a cross-sectional design, and clinical translation has been poor. In this study, we investigated the longitudinal associations between NAFLD and SNPs in the Korean population. Although we did not see any genome-wide significant associations with the development or regression of NAFLD that met the Bonferroni correction criteria, the present study has a role as an exploratory study. Previously, we performed a GWAS with a cross-sectional design and found that the *PNPLA3* and *SAMM50* genes are significantly associated with NAFLD, even after adjusting for age, sex and BMI, in the Korean population [12]. Considering these results, the presence of NAFLD is significantly associated with SNPs, while longitudinal changes in NAFLD, such as development or regression, may not be associated with genetic variants.

*PNPLA3* is a well-known genetic variant that is associated with NAFLD, and the severity of steatohepatitis and fibrosis has been validated in various ethnic groups via GWAS [20, 21]. A recent GWAS showed that four genetic variants (s759359281 in *SLC30A10*, rs13107325 in *SLC39A8,* rs58542926 in *TM6SF2*, and rs738409 in *PNPLA3*) had variable effects on liver fat measured using MRI and other metabolic traits [22]. The pathogenic mechanism through which the *PNPLA3* variant contributes to NAFLD development and progression has been extensively investigated. In brief, *PNPLA3* is involved in lipid metabolism and modulates the accumulation of hepatic triglycerides [23, 24]. In an exome-wide study, impaired function of transmembrane 6 superfamily member 2 (*TM6SF2*) promoted NAFLD by reducing very low-density lipoprotein secretion [25]. In a histologically confirmed case-control study, rs58542926, located in the *TM6SF2* locus, was a low-frequency variant with a modest effect on NAFLD, even after conditioning on *PNPLA3*-rs 738409 and metabolic risk factors [26]. In addition, the membrane bound O-acyltransferase domain-containing 7 (*MBOAT7*) gene was associated with the risk of NAFLD in the European Caucasian population [27]. A GWAS identified variants in the *MBOAT7* and *TM6SF2* genes as new risk loci for alcohol-related cirrhosis [28]. Recently, it has been found that the association of rs6834314 with ALT reflects its association with NAFLD and that 17-beta hydroxysteroid dehydrogenase 13 (HSD17B13) plays a role in NAFLD through its enzymatic activity [29]. In addition, combined effects of multiple genetic variants on NAFLD severity were suggested in a multicenter biopsy-based study [30]. However, all of these studies were based on a cross-sectional design; thus, knowledge of the longitudinal effects of genetic variants on the development or regression of NAFLD is limited.

Several studies have been conducted to investigate whether genetic variants influence changes in clinical characteristics. A previous study regarding the effect of the rs738409 *PNPLA3* allele on the ability of weight loss to decrease liver fat showed that hepatic fat decreased by 45% in the *PNPLA3*-148MM group and by 18% in the *PNPLA3*-148II group, suggesting a role of rs738409 in lifestyle modification [31]. However, this study did not evaluate fatty liver as a variable, unlike our research. In a single-blind randomized controlled trial, the researchers correlated the *PNPLA3* rs738409 gene polymorphism with changes in metabolic profile and intrahepatic triglycerides. Although the presence of the G allele in the *PNPLA3* rs738409 gene polymorphism was associated with a greater reduction in metabolic parameters, including intrahepatic triglycerides, body weight, the waist-to-hip ratio, blood total cholesterol, and low-density lipoprotein levels, there was no significant difference in the remission of NAFLD among patients with different *PNPLA3* rs738409 genotypes [32]. Consistent with the previous study, we did not reach the threshold for significance in this study; only 1 SNP (rs4906353) in the gene that encodes kinesin light chain 1 (KCL1) on chromosome 14 showed an association with the development of NAFLD, with marginal significance; however, this SNP did not pass the Bonferroni correction for genome-wide significance.

One explanation for this result may be related to the pathogenesis of NAFLD development. The development or regression of NAFLD is determined by a combination of environmental factors, such as gut microbiota and dietary components, and multiple genetic factors [33]. Indeed, hepatic steatosis-related genetic variants are associated not only with nonalcoholic steatohepatitis or fibrosis but also abnormal metabolic traits, including serum lipids, glucose and anthropometric measures [34]. In addition, the *PNPLA3* genotype has been elucidated as a modifier of NAFLD-associated metabolic systemic diseases such as carotid atherosclerosis [35] and chronic kidney disease [36]. However, the effect of genetics via SNPs may be slow and could be compensated by various factors, and changes in NAFLD may take longer than we expected.

Another possible explanation is the decreased power caused by the smaller sample size. While the threshold of *P*-value < 0.05 is considered to be statistically significant in conventional observational studies, GWAS results have much smaller *p*-values. For GWAS, the genome-wide significance threshold has usually been suggested to be a *P*-value < 5 × 10^− 8^ [37]. Since a larger sample size in an association study has a higher chance of having a statistical significance, a genetic association that is found to be significant in its initial GWAS is generally replicated in multiple studies. Thus, complex interactions, including the characteristics of the study population, the types of genetic data or arrays used for the analysis, minor allele frequencies of SNPs, and different patterns of linkage disequilibrium, should be taken into account when interpreting the GWAS data [38]. To confirm the longitudinal association of NAFLD with genetic variants, future studies with larger sample sizes are needed.

Although this study did not demonstrate a significant association between SNPs and the development or regression of NAFLD, it is the first study to investigate the longitudinal association between SNPs and the risk of NAFLD in an apparently healthy population that presented for health check-ups. This study has several limitations. First, the population in this study was based on subjects who voluntarily underwent health check-ups at a single center in Korea. It may not be representative of the general population, and there could be regional and economic selection bias compared with the entire Korean population. Second, the validation set was recruited from the same single-center population as the discovery set. Since some subjects from the same population were sampled in both sets, the selection of subjects may affect the results of this study. In the future, a validation study should be performed using a different population set recruited from a different location. Third, although genetic determinants affect changes in NAFLD, the development or regression of NAFLD is largely associated with the worsening or improvement of metabolic traits, which is related to residual confounding factors, such as lifestyle modifications, changes in medical treatment or health status, and could not be evaluated in this study. Since the effect of lifestyle and diet may surpass the effect of SNPs, further study is needed to take into account changes in lifestyle and diet to confirm the actual association of SNPs with the development of NAFLD. Fourth, although the effect of genetics via SNPs may be slow and could be compensated by various factors, and the median follow-up duration in this study may be too short to investigate the change in NAFLD. When genetic variants affect the development of NAFLD, a long-term follow-up period is necessary. Finally, although ultrasonography for the NAFLD diagnosis is an operator-dependent procedure and not sensitive enough to detect minor steatosis, histological changes using liver biopsy could not be evaluated in the present study.

## Conclusions

This exploratory study demonstrated that longitudinal changes in NAFLD are not associated with genetic variants in the Korean population. These findings provide new insight into the genetic mechanisms involved in the pathogenesis of NAFLD. Since the present study was limited to a relatively small sample size of Korean ethnicity, further studies are needed to confirm the results in a larger number of participants.

## Supplementary information

**Additional file 1:** **Figure S1.** Manhattan plot of genome-wide association signals between SNPs and NAFLD regression.

## Data Availability

The datasets used and/or analyzed during the current study are available from the corresponding author on reasonable request.

## Abbreviations

NAFLD: Nonalcoholic fatty liver disease
GWAS: Genome-wide association study
SNP: Single-nucleotide polymorphism
PNPLA3: Patatin-like phospholipase domain-containing protein 3
BMI: Body mass index
AST: Aspartate aminotransferase
ALT: Alanine aminotransferase
HDL: High-density lipoprotein
HR: Hazard ratio
CI: Confidence interval
