# Supplementary figures and images for: The association of genetic polymorphisms with nonalcoholic fatty liver disease in a longitudinal study
Source: BMC Gastroenterol. 2020 Oct 15;20:344. doi: 10.1186/s12876-020-01469-8 (PMC7565807; doi:10.1186/s12876-020-01469-8)

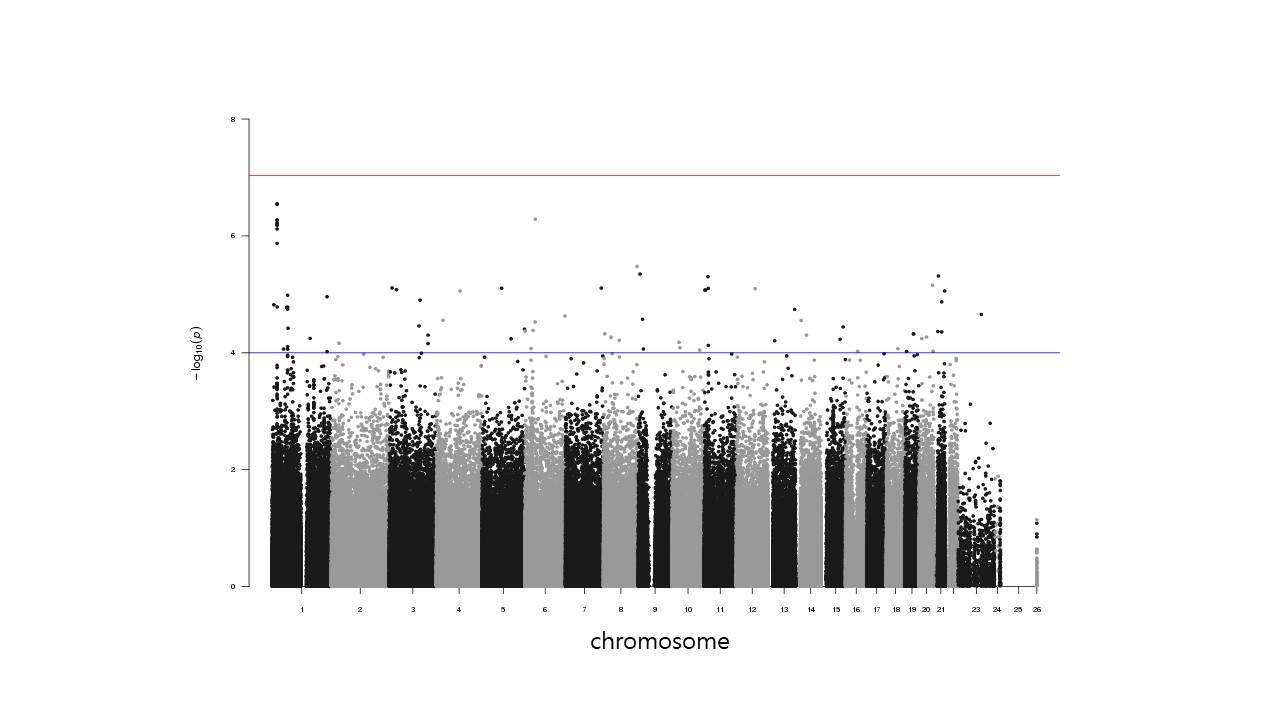

Supplement: Supplementary file 1 — Additional file 1: Figure S1. Manhattan plot of genome-wide association signals between SNPs and NAFLD regression. [file 12876_2020_1469_MOESM1_ESM.jpg]
